# Protection against lung pathology during obesity-accelerated ageing in mice by the parasitic worm product ES-62

**DOI:** 10.3389/fimmu.2023.1285069

**Published:** 2023-11-23

**Authors:** Margaret M. Harnett, Felicity E. Lumb, Jenny Crowe, James Doonan, Geraldine Buitrago, Stephanie Brown, Gillian Thom, Amy MacDonald, Colin J. Suckling, Colin Selman, William Harnett

**Affiliations:** ^1^ School of Infection and Immunity, University of Glasgow, Glasgow, United Kingdom; ^2^ Strathclyde Institute of Pharmacy and Biomedical Sciences, University of Strathclyde, Glasgow, United Kingdom; ^3^ Department of Pure & Applied Chemistry, University of Strathclyde, Glasgow, United Kingdom; ^4^ School of Biodiversity, One Health and Veterinary Medicine, University of Glasgow, Glasgow, United Kingdom

**Keywords:** ageing, allergy, helminth, immunomodulation, lung pathology, obesity, Th2

## Abstract

Mice develop pathology in the lungs as they age and this may be accelerated by a high calorie diet (HCD). ES-62 is a protein secreted by the parasitic worm *Acanthocheilonema viteae* that is immunomodulatory by virtue of covalently attached phosphorylcholine (PC) moieties. In this study, we show that weekly treatment of C57BL/6J mice with ES-62 protected against pathology in the lungs in male but not female mice fed a HCD from 10 weeks of age as shown by reductions in cellular infiltration and airway remodelling, particularly up to 160 days of age. ES-62 also reduced gene expression of the cytokines IL-4 and IL-17 and in addition the TLR/IL-1R adaptor MyD88, in the lungs of male mice although HCD-induced increases in these inflammatory markers were not detected until between 340 and 500 days of age. A combination of two drug-like ES-62 PC-based small molecule analogues (SMAs), produced broadly similar protective effects in the lungs of male mice with respect to both lung pathology and inflammatory markers, in addition to a decrease in HCD-induced IL-5 expression. Overall, our data show that ES-62 and its SMAs offer protection against HCD-accelerated pathological changes in the lungs during ageing. Given the targeting of Th2 cytokines and IL-17, we discuss this protection in the context of ES-62’s previously described amelioration of airway hyper-responsiveness in mouse models of asthma.

## Introduction

Over the past few decades, there has been much interest in the idea that parasitic worm infection might prevent people from developing allergic conditions such as asthma [reviewed in (1)]. This has resulted in the anti-allergy properties of parasitic worms being routinely investigated in mouse models and the general success of this approach has led to researchers setting out to identify the active molecules secreted by the worms and their mechanism of action [reviewed in (2)]. One such molecule is ES-62, a phosphorylcholine (PC)-containing glycoprotein, produced by the rodent filarial nematode *Acanthocheilonema viteae* [reviewed in (3)]. This molecule can prevent mice from developing ovalbumin (OVA)-induced airway hyper-responsiveness in both acute and chronic models and reduce airway remodelling in the latter (4–6). Its mechanism of action is multifold and includes blocking of mast cell degranulation, preventing eosinophil and neutrophil infiltration of the lungs (4–6), inhibiting Th2 and Th17 responses via counter-regulation by IFNγ (5), and normalising diminished levels of regulatory B cells (Bregs) (6).

ES-62 has also recently been tested in a C57BL/6J mouse model of obesity-accelerated ageing (7, 8). The logic underlying this study is that chronic low grade inflammation is recognised as a hallmark of ageing (9) and one that is augmented and accelerated by a high calorie diet (HCD) [reviewed in (10)]. It was hypothesised that as an anti-inflammatory agent, ES-62 might counter this. One of the traits that emerged from this study was that the HCD-fed mice developed pathological changes across a wide range of organs as they aged and that ES-62 could often protect again this, particularly in male animals (7, 8). Of note, contrary to previous views that asthma is a disease of the young it is now recognised to be as prevalent in the elderly but is often under- or mis-diagnosed [reviewed in (11)]. Furthermore, it is known that obesity can significantly enhance the severity of asthma [reviewed in (12)]. Thus, given ES-62’s ability to protect against disease in the lung during asthma it was decided to investigate whether lung pathology was apparent in the obesity-accelerated ageing model and if so, whether the helminth-derived molecule could protect against this.

## Materials and methods

### Mouse model of obesity-accelerated ageing

**Figure 1:**
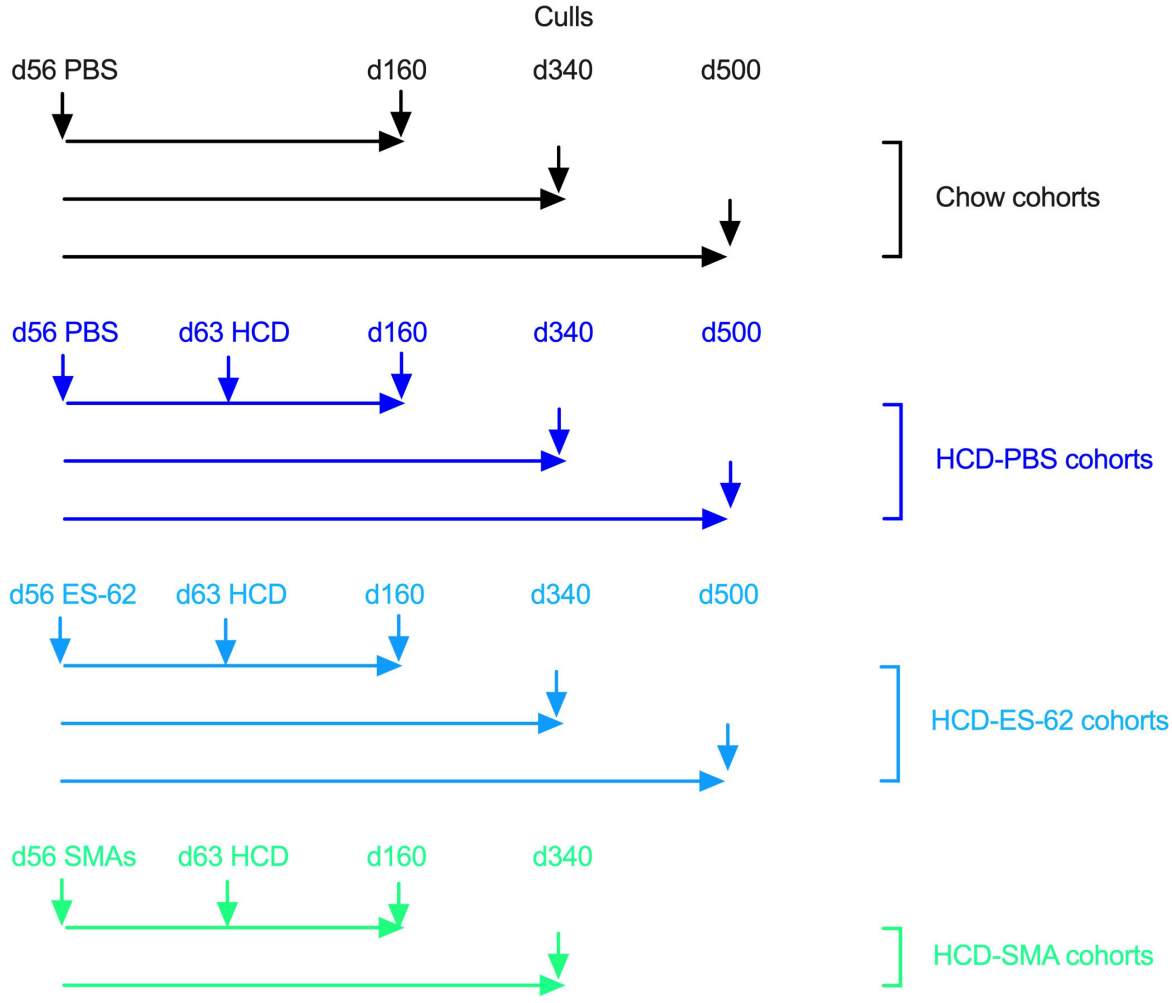
Schematic describing the time-course and interventions of Chow and HCD-fed mouse cohorts.

Male and female C57BL/6J mice (Envigo, UK) were housed in the Central Research Facility, University of Glasgow and maintained, under specific pathogen-free conditions, at 22°C under a 12-h light/dark cycle with *ad libitum* access to water and Chow (CRM-P) or High Calorie (Western Diet RD) diets from Special Diet Services, UK as described previously (7, 8, 13). All procedures were performed in accordance with UK Home Office Project Licences (60/4504 and PDBDC/7568), following the “principles of laboratory animal care” (NIH Publication No. 86-23, revised 1985) and approval by the University of Glasgow Animal Welfare and Ethical Review Board. Briefly, mice were randomly allocated to groups on arrival at 4 weeks of age (to same sex cages of 2 to 4) and fed normal Chow CRM-P diet (comprising Oil, 3.36%; Protein 18.35%; Fibre, 4.23%: Sugar 3.9%; Atwater fuel energy from Oil, 9.08%; Protein, 22.03%: Carbohydrate, 68.9%) plus 150 ppm Fenbendazole. From 9 weeks of age (**Figure 1**), mice were administered (subcutaneously) PBS, purified endotoxin-free ES-62 (1 μg) or a combination of SMAs 11a plus 12b (both 1 µg) weekly and then, at 10 weeks of age, the HCD cohorts received Western Diet RD (Fat, 21.4%; Protein, 17.5%; Fibre, 3.5%; Sucrose 33%; Atwater fuel energy from Fat, 42%; Protein, 15%: Carbohydrate, 43%) plus 150 ppm Fenbendazole. Mice were culled as indicated at 56 (prior to HCD/ES-62 treatment), 160 (young age point), 340 (mid age point) and 500 (old age point; ES-62 study only) days of age via cervical dislocation, with whole lungs being harvested. The right lobes of the lung were transferred to 4% paraformaldehyde for fixation for histology, and the left lobe was flash frozen prior to mRNA extraction for RT-PCR analysis.

### Lung histology

Following fixation, the right lobes of lungs were embedded in OCT and stored at -80°C until preparation of 7-10 µm sections (Shandon Cryotome Cryostat; Fisher Scientific) affixed onto Thermo Scientific™ SuperFrost™ Plus Adhesion slides. For Haematoxylin and Eosin (H&E) staining, sections were treated with ice cold 75% acetone:25% ethanol solution for 10 minutes, then rehydrated in PBS solution for 25 minutes. The sections were then placed in Haematoxylin solution (Sigma Aldrich, Poole, UK) for 10 minutes, washed in running tap water and dipped into 1% hydrochloric acid:70% ethanol prior to washing in Scott’s tap water solution (3.5g sodium bicarbonate, 20g magnesium sulphate in 1L dH_2_O) until a blue colouration was observed. Counterstaining of acidophilic structures was achieved by treating with Eosin solution (Sigma Aldrich, Poole, UK) for 4 minutes. Following washing in running tap water, sections were dehydrated via exposure to increasing concentrations of ethanol (70%, 90% and 100%), then fixed in Histoclear solution (National Diagnostics, Atlanta, Georgia) for one minute. DPX mounting medium (Sigma Aldrich) was used to mount glass cover slips over the samples. H&E staining was then analysed using an EVOS FL Auto Cell Imaging System (Thermofisher Scientific) at 20x magnification, with sections scored blindly for cellular infiltration and airway remodelling (epithelial/goblet cell hyperplasia and smooth muscle alteration). Specifically, scoring was performed on a 1 to 5-point scale for each of these parameters where, 1= normal morphology, 2= mild pathology with limited alteration to tissue structures, 3= moderate pathology and alteration of tissue structures, 4= substantial pathology but some tissue structures remaining and 5=severe pathology and remodelling of tissue structures. An “overall” pathology score was then generated for each sample by taking the sum of the scores acquired across all these parameters and then averaging it.

### Quantitative reverse transcriptase polymerase chain reaction

For RNA Extraction, frozen lung samples were homogenised in QIAzol^®^ Lysis Reagent (Qiagen, Hilden, Germany) using a TissueRuptor (Qiagen). Chloroform was added to the tissue lysate, which was then centrifuged for 15 minutes (12,000 x g at 4°C) and genomic (g)DNA removed from the aqueous phase using gDNA eliminator columns. RNA purification was carried out using EZ-10 DNAaway RNA Mini-Preps Kit (Bio Basic Canada Inc., Ontario, Canada) according to the manufacturer’s handbook. RNA levels were then measured via an Epoch/BioTek system using the GEN5.1.10 program, and 2000 ng of RNA was reverse transcribed to cDNA using the High Capacity cDNA Reverse Transcriptase kit (Applied Biosystems, Foster City, US) using a Veriti Thermal Cycler (Applied Biosystems). cDNA was amplified using the StepOne Plus™ real-time PCR system (Applied Biosystems), and SYBR ^®^ Green KiCqStart^®^ SYBR^®^ green qPCR ReadyMix with ROX™ and pre-designed KiCqStart^®^ primers (all Sigma Aldrich) using 20 ng cDNA per triplicate sample. Target gene expression was normalised to the housekeeping control β-actin (Actb) and RQ values (2^-ΔCT) calculated. Forward and reverse primers (5’-3’) were respectively: Actb, GATGTATGAAGGCTTTGGTC and TGTGCACTTTTATTGGTCTC; MyD88, TAATGAGAAAAGGTGTCGC and ATACTGGGAAAGTCCTTCTTC; IL-4, CTGGATTCATCGATAAGCTG and TTTGCATGATGCTCTTTAGG; IL-5, CCCTACTCATAAAAATCACCAG and TTGGAATAGCATTTCCACAG; IL-17, ATTCAGAGGCAGATTCAGA and AACAAACACGAAG CAGTT; IFNγ, TGAGTATTGCCAAGTTTGAG and TTATTGGGACAATCTCTTCC.

### Statistics

GraphPad Prism 10 software was used to analyse all data obtained, and data presented as mean values for individual mice ± SEM. One and Two-way ANOVA tests with Fisher’s LSD test and non-parametric Kruskal Wallis tests were performed, with statistical significance being indicated at *p<0.05, **p<0.01, ***p<0.001 and ****p<0.0001.

## Results

### ES-62 protects against ageing-associated lung pathology in male mice

**Figure 2:**
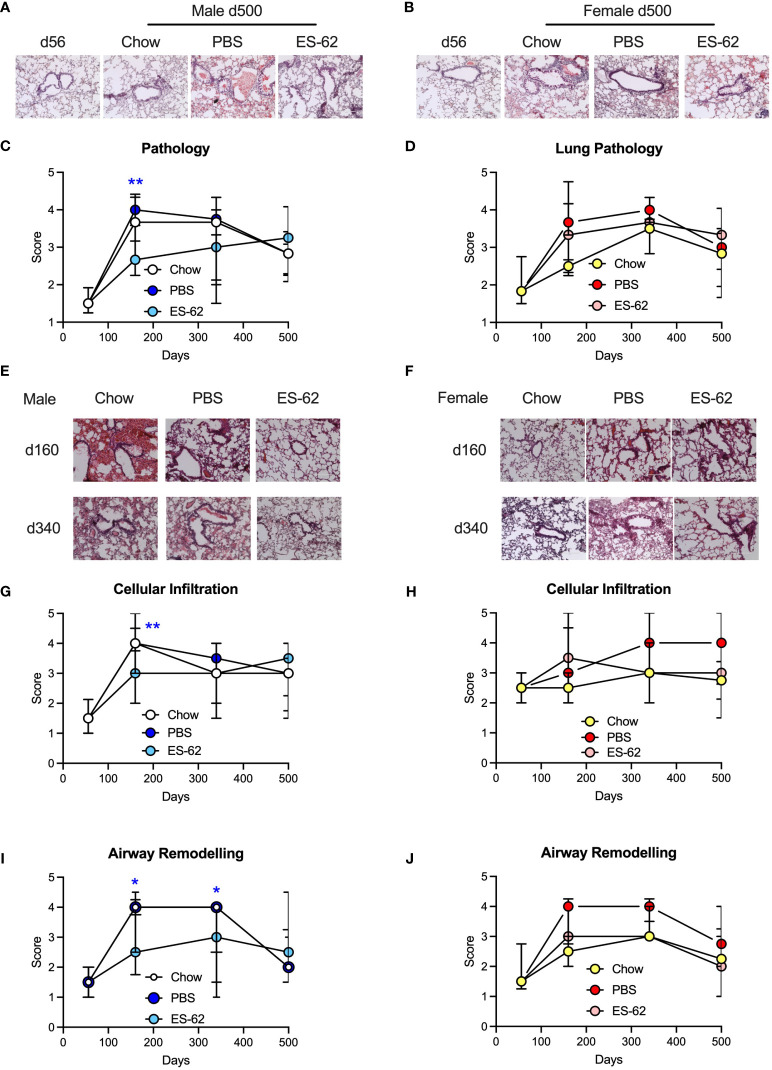
ES-62 delays lung histopathology associated with ageing in male mice. Representative images of H & E staining of lung tissue from male **(A)** and female **(B)** mice fed Chow (d56 and d500 Chow) or HCD, the latter treated with either PBS or ES-62 (d500 PBS and d500 ES-62). Semi-quantitative analysis (scoring) of lung pathology in male **(C)** and female **(D)** mice over the time-course of the experiment where the data are presented as the median values of the treatment group ± interquartile range, where n=individual mice. Representative images of H & E staining of lung tissue from male **(E)** and female **(F)** mice fed Chow or HCD, the latter treated with either PBS or ES-62 (PBS and ES-62) at 160 and 340 days of age. Semi-quantitative analysis (scoring) of cellular infiltration of the lung **(G, H)** and airway remodelling **(I, J)** in male **(G, I)** and female **(H, J)** mice over the time-course of the experiment where the data are presented as the median values of the treatment group ± interquartile range. Images **(A, B, E, F)** were taken at x20 magnification using an EVOS cell imaging system (scale bars of 200 µm). For histological scoring **(C, D, G-J)** the numbers of individual mice were: males, Chow: d56 n=6; d160 n=5; d340 n=4; d500 n=5; PBS: d160 n= 10; d340 n=6; d500 n=6; ES-62: d160 n= 9; d340 n=9; d500 n=4; females, Chow: d56 n=5; d160 n=2; d340 n=6; d500 n=4; PBS d160 n=9; d340 n=7; d500 n=6; ES-62: d160 n= 10; d340 n=10, d500 n=5. Statistical analysis was by two-way ANOVA and where *p<0.05 and **p<0.01 for the ES-62 v PBS groups.

Mice were initially fed on a normal chow diet and were given weekly administration of ES-62 (1 µg/dose) or a PBS control from 9 weeks of age. At 10 weeks of age the indicated cohorts of animals (PBS or ES-62) were switched to HCD with some being retained on chow (Chow; PBS only) to serve as a control for HCD feeding. Analysis of lung tissue from 500 day old mice showed evidence of pathology (relative to young d56 mice) in all cohorts of male and female mice (**Figures 2A, B**). When lungs were examined from animals culled at earlier time points, it was found that such pathology had appeared between d56 and d160 of age and was maintained at elevated levels, particularly in female mice, over the 500-day time course although with a gradual age-related decrease in both Chow and HCD-fed animals (**Figures 2C-F**). ES-62 offered significant protection against lung pathology in HCD-fed mice when assessed at 160 days of age, although this was only seen in male animals (**Figures 2C-F**). Further analysis revealed that this protection was associated with reduced cellular infiltration in the ES-62-treated male but not ES-62-treated female animals (**Figures 2G, H**). Interestingly, the male Chow and HCD (PBS)-fed mice showed a stronger, more transient cellular influx than the female mice with both appearing to show, albeit non-significantly, HCD-enhanced effects on this parameter at d340 (**Figures 2G, H**). Whilst the anti-inflammatory action of ES-62 in male mice was lost by 340 days of age, administration of the helminth product offered protection against HCD-induced airway remodelling in both d160 and d340 male, but not female mice (**Figures 2I, J**).

**Figure 3:**
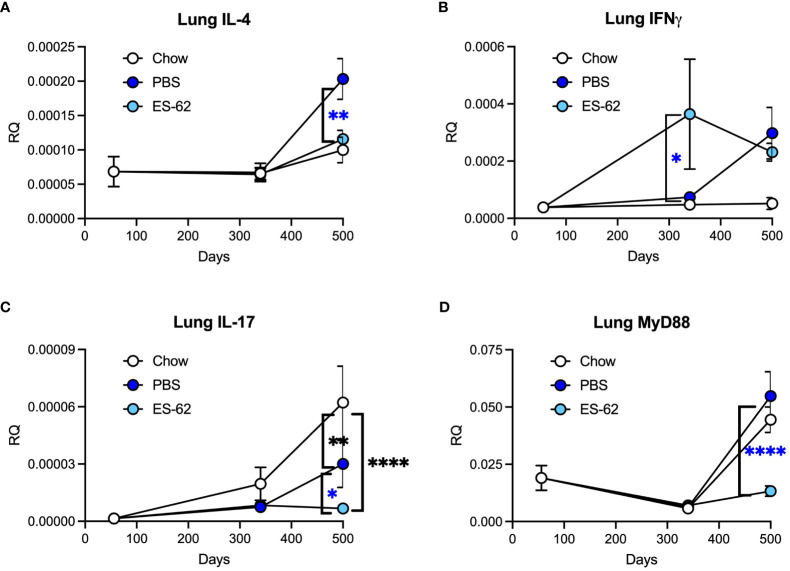
ES-62 modulates the expression of inflammatory markers in the lungs of ageing male mice. Expression of IL-4 **(A)**, IFNγ **(B)**, IL-17 **(C)** and MyD88 **(D)** in lung tissue at the mRNA level in mice over the time-course of the experiment where the data are presented as the mean values of the treatment group ± SEM, where n=individual mice. The numbers of mice tested were: Chow: d56 n=6; d340 n=6; d500 n=5; PBS: d340 n=7; d500 n=6; ES-62: d340 n=8 (n=7 for IL-4); d500 n=6. Statistical analysis was by two-way ANOVA and where blue *p<0.05 **p<0.01 and ****p<0.0001 for the ES-62 v PBS groups and black **p<0.01 and ****p<0.0001 for the Chow group v PBS and ES-62 groups, respectively.

RT-PCR analysis of inflammatory mediators in lung tissue from male mice was carried out for evidence of potential changes that could help explain the ES-62-mediated protection observed in histological analysis. HCD increased expression of IL-4, not by day 160 as was observed for lung pathology, but at a later time point between day 340 and day 500 (**Figure 3A**). ES-62 provided significant protection against this increase, returning the cytokine level to a value comparable to that associated with Chow feeding (**Figure 3A**). HCD also increased expression of IFNγ by day 500 in male animals (**Figure 3B**). ES-62 had no effect on this but did significantly raise levels of this cytokine at day 340. HCD reduced expression of IL-17 between day 340 and day 500 in male animals and ES-62 diminished it further (**Figure 3C**). Levels of the known ES-62 target, MyD88 (3), increased greatly in both chow-fed and HCD-fed male mice between day 340 and day 500 and ES-62 was extremely effective in preventing this when administered to male animals fed HCD (**Figure 3D**).

### ES-62 small molecule analogues protect against ageing-associated lung pathology in male mice

Many aspects of the protective effects of ES-62 in models of OVA-induced hypersensitivity (6, 14) and also obesity-accelerated ageing (8, 13) can be mimicked by each of two drug-like small molecule analogues (SMAs), 11a and 12b. The two SMAs are modelled on ES-62’s active PC moiety but in slightly different ways. One is a tertiary amine (11a) and the other a quaternary ammonium salt (12b), giving them subtly different chemical properties which leads to some differences in biological profile, e.g., with respect to targeting of individual pro-inflammatory cytokine species (14–16). Similarly to ES-62, the effect of a combination of the two SMAs on the obesity-accelerated ageing model was thus investigated in male mice. Mice were initially fed on chow and were given weekly administration of a cocktail of SMAs 11a and 12b (1 µg each/dose) or a PBS vehicle control from 9 weeks of age. Again, at 10 weeks of age cohorts were switched to HCD (PBS or SMAs) with some being retained on chow (Chow; PBS only) to serve as ageing controls for HCD feeding and lungs were examined from male animals culled at each of day 56, 160 and 340.

**Figure 4:**
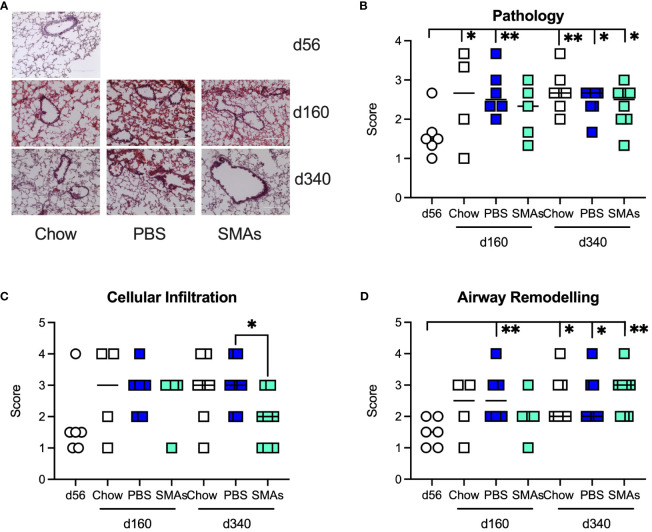
ES-62 SMAs delay lung histopathology associated with ageing in male mice. **(A)** Representative images of H & E staining of lung tissue from male mice fed Chow (d56, d160 and d340) or HCD, the latter treated with either PBS or ES-62 SMAs 11a and 12b (PBS, d160 and d340; ES-62 SMAs, d160 and d340). Semi-quantitative analysis (scoring) of lung pathology **(B)**, cellular infiltration **(C)** and airway remodelling **(D)** over the time-course of the experiment where the data are presented as the mean values of replicate images, where symbols denote the individual mice and the bars represent the mean values of the treatment groups. The numbers of individual mice were Chow: d56 n=6 (same data as in **Figure 2**); d160 n=4; d340 n=7; PBS: d160 n= 6; d340 n=8; SMAs: d160 n= 5; d340 n=8. Statistical analysis was by one-way ANOVA and where *p<0.05 and **p<0.01 for the d56 v indicated groups or for **(C)** PBS v SMAs groups at d160 and/or d340.

Analysis of histopathology revealed data broadly in keeping with the earlier study focusing on ES-62, as there were significant increases in pathology in the lungs of both Chow and HCD (PBS)-fed mice relative to day 56 mice. There was some evidence for the SMAs protecting against this at least at day 160 as the value for HCD-fed mice treated with the SMAs was not significantly different from the day 56 basal value (**Figures 4A, B**). Likewise, the SMAs also reduced cellular infiltration (**Figure 4C**), significantly at d340, and airway remodelling at day 160 (**Figure 4D**).

**Figure 5:**
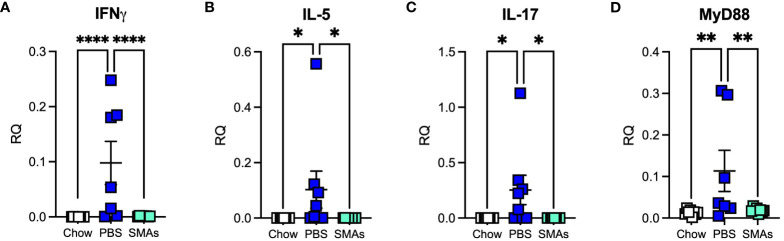
ES-62 SMAs modulate the expression of inflammatory markers in the lungs of ageing male mice. Expression of IFNγ **(A)**, IL-5 **(B)**, IL-17 **(C)** and MyD88 **(D)** in lung tissue at the mRNA level in mice at day 340 where the data are presented as the mean RQ values for individual mice (amplifications performed in triplicate) and the bars represent the mean values ± SEM of the treatment groups. The numbers of individual mice are: Chow: n=8; PBS: n= 7 (IFNγ, MyD88) or 8 (IL-5, IL-17); SMAs: n=8. Statistical analysis was by one-way ANOVA and significant differences between the PBS v Chow and PBS v ES-62 groups as indicated and where *p<0.05 **(B, C)**, **p<0.01 **(D)** and ****p<0.0001 **(A)**.

RT-PCR analysis of lung tissues in this experiment, unlike in the ES-62 experiment revealed HCD-induced changes in certain cytokines by day 340. Thus, IFNγ, IL-17 and IL-5 were significantly increased by day 340 and the administration of the SMA cocktail was found to return all of these to Chow levels (**Figures 5A-C**). Consistent with the ES-62 experiment, no increase in IL-4 was observed at day 340 (result not shown). MyD88 was also elevated in the HCD group at this time point in this experiment and again the combination of the two SMAs, which also target this key inflammation transducer (17), prevented this increase (**Figure 5D**).

## Discussion

It is known that as with other body organs, the lungs become less efficient with age and indeed ultimately develop a high basal inflammation profile in both humans and mice (18). Furthermore, obesity may also contribute to impaired lung function (19). It was therefore not surprising that as with a series of other body organs (7), ageing was associated with pathological changes in the lungs in our study as shown by histological examination. The effect was observed in both male and female animals and while HCD administration had no effect on the emergence of pathology in male animals, it accelerated pathology in female animals. ES-62 did not mitigate lung pathology in female mice but significantly reduced it, particularly with respect to cellular infiltration, in male animals at the day 160 time-point when pathology was at its highest level in both chow- and HCD-fed animals. Although we have not previously compared the effect of gender on ES-62 activity in the lungs in asthma models, we have found it to vary with respect to protective effects between male and female HCD-fed mice in other organs, for example, the gut (7) and bone (8) where protection is seen in male but not female animals. However, our previous studies on models of acute (5) and chronic (6) ovalbumin-induced airway hyper-responsiveness actually employed female mice, showing that ES-62-protection against lung disease is not restricted to male animals but rather appears to be model-dependent. The absence of ES-62-driven protection in male mice at later time-points in the current study may reflect reduced lung pathology and cellular infiltration dictating that there was less pathology for the helminth-derived molecule to work on. This result is consistent with our previous observation that some organ/cell systems in HCD-fed mice actually show some signs of improvement in the absence of treatment as the animals move from 340 to 500 days of age (7). Nevertheless, ES-62 was able to protect against HCD-induced airway remodelling at both the day 160 and day 340-timepoints in male animals. Of note, previously ES-62 has been shown to protect against airway remodelling in a model of ovalbumin-induced chronic asthma and once again that was undertaken in female mice (6). We have not yet fully established the reason for sex-specific effects with respect to ES-62 activity in mouse models of human diseases but in addition to the likely involvement of hormones, our previous work (7) suggests the involvement of a different gut microbiome composition.

The ability of ES-62 to reduce pathology in the lungs of male mice led us to examine male lung tissue for ES-62 induced changes in expression of genes related to the inflammatory response. Animals were found to have significantly increased IL-4 gene expression in the lungs but this did not occur until after 340 days of age and was found to be dependent on HCD consumption. ES-62 significantly protected against the increase in IL-4 expression returning levels to those associated with a chow diet. IL-4 is of course a signature Th2 cytokine that plays a role in asthma and indeed, ES-62 has been previously shown to inhibit IL-4 responses in the acute ovalbumin-induced airway hyper-responsiveness model. The mechanism underlying ES-62’s action against IL-4 in this model is associated with reversal of T helper cell polarity from Th2 to Th1 and in particular, increased production of IFNγ (5). Levels of this cytokine were elevated by day 340 in ES-62-treated HCD-mice but also increased to approximately the same level in PBS-treated HCD-mice between days 340 and 500. Thus, it cannot be said with certainty whether in the present study, ES-62’s ability to reduce IL-4 levels reflects increased IFNγ expression. As with our earlier asthma study (5), this could be investigated using anti-IFNγ antibody treatment. Our previous asthma study also showed that ES-62 targeted production of IL-17 (5), a cytokine that can be pathogenic in asthma and similarly in the present study the helminth product reduced lung expression of this cytokine in HCD-treated mice by day 500. However, HCD-treated animals administered PBS also revealed lower levels of expression than chow mice suggesting that this cytokine rises during ageing even in the absence of HCD-feeding. Of note, the reduction in IL-17 in response to HCD may be related to the simultaneous increase in IL-4 as the latter cytokine is known to inhibit production of the former (20). There was also an age-associated increase in expression of the TLR/IL-1R adaptor MyD88 in lung tissue from both chow-fed and HCD-fed mice and this appeared between day 340 and day 500. ES-62 dramatically attenuated this increase in the HCD-fed cohort and this result is consistent with its effect on MyD88 expression in lung tissue from mice subjected to the chronic model of ovalbumin-induced airway hyper-responsiveness (6) and also in both mouse Th17 cells (21) and mast cells (22).

As ES-62 drug-like SMAs 11a and 12b mimic its mechanism of action in several models of diseases associated with inflammatory responses including lung allergy (6, 14) and non-lung parameters of obesity accelerated ageing (13), these compounds were also tested in the present study for their effect on the lungs of male animals. Similar to the ES-62 experiment, there was some evidence for the combination of SMAs offering a degree of protection against pathology associated with HCD-accelerated ageing at the day 160 time-point. In addition, the SMAs demonstrated inhibition of airway remodelling at this time-point and reduced cellular infiltration at day 340. Moreover, we observed significant increases in the cytokines IFNγ, IL-5 and IL-17 and in addition MyD88, following administration of HCD but not chow diet. Such increases appeared by day 340, which was earlier than we had seen for the appearance of inflammatory markers in the ES-62 study. As both studies were carried out using the same animal house, mouse strain and HCD, there is no obvious explanation for this but it may be that we are only dealing with a difference of a few days or weeks in time (measurements were only made at days 160 and 340) and it is possible that transient microbiome composition differences could play a role as our previous work showed that cytokine changes in various tissues were highly dynamic (7). Unlike with the SMA study, increases in IL-17 and MyD88 were seen in the ES-62 study when employing the chow diet but this was at a time-point (day 500) beyond the termination point of the SMA study (day 340). Treatment with the SMAs reduced all four HCD-induced increases in gene expression in lung tissue to levels associated with chow feeding. The decreases in IL-17 and MyD88 mirrored what we observed in the ES-62 study. However, IFNγ was increased at this time-point in the ES-62 study and this is consistent with what we find in allergy models with respect to both the parent molecule (5) and its SMAs (14), where their mechanism of action is considered to at least in part reflect a reversal of Th2 polarisation. Finding a difference between the actions of ES-62 and its SMAs is unusual in our experience as usually both are protective [e.g (5, 6, 14)] or not (23) in a particular disease model. Also, unlike with our allergy models where increasing IFNγ offers protection against disease, our data in the current study do not offer definitive evidence on whether the cytokine has protective or pathological (or both) roles in the lungs. We have not previously measured IL-5 when testing the SMAs in allergy models but ES-62 has previously been shown to reduce mRNA levels in draining lymph nodes in the acute OVA model (5). Finally, as with the ES-62 study, IL-4 was not detected at day 340 in the SMA study.

The cells contributing to the observed pathology – cellular infiltration, airway remodelling, cytokine production - in the HCD-fed mice as they age, and which are targeted by ES-62 and the SMAs, have not been determined. The situation is likely to be complex as our earlier work on the model, revealed not only that many immune system cell types vary in quantity as the mice age but also that ES-62 only impacts on the levels of these cell populations e.g., eosinophils in gonadal fat, Bregs in the mesenteric lymph nodes, at certain time points during ageing (7). Focusing on the lungs, we noted similar dynamic changes in the levels of these two cell populations during the chronic model of ovalbumin-induced airway hyper-responsiveness (6). Our previous work in fact indicates that ES-62 interacts with many cell types (reviewed in 2 and 3) and indeed it is likely that it can target any cell that expresses TLR4. The same seems to be true of the SMAs and indeed these compounds may even pass across the plasma membrane in the absence of TLR4 due to their small size and particular chemical structures. Certainly, we have seen effects of ES-62 and/or the SMAs on a wide range of cells including B cells, T cells, macrophages, dendritic cells, mast cells, fibroblasts, neutrophils, eosinophils and Bregs (see for example references 2 and 3) and most of these with respect to asthma where ES-62 and the SMAs have been shown to prevent infiltration by cell types such as eosinophils, neutrophils, lymphocytes and mast cells (4–6). This suggests there is also a number of options for the identity of the cells being targeted by ES-62/SMAs in the lungs with respect to cytokine production. Overall, it is perhaps likely that the data we present in the current study may, rather than the targeting of a particular cell-type, reflect complex manipulation of a cellular network as we proposed for ES-62’s modulation of the dynamic interactions of neutrophils, mast cells and Bregs during allergic exacerbations in the ovalbumin-induced chronic asthma model (6).

In summary, although some of the mechanistic detail remains to be elucidated, for example, with respect to the role of targeting individual cytokines like IFNγ, overall this study clearly shows that both the parasitic worm product ES-62 and its drug-like SMAs, can protect against lung pathology and inflammation during obesity-accelerated ageing in mice. Although a number of helminth-derived molecules other than ES-62 can protect mice against the development of allergy in the lungs [reviewed in (2)], this is the first study as far as we are aware to show protection in the lungs in a model of obesity-accelerated ageing. It is interesting that we see increases in IL-4 and IL-5 in the lungs of animals in the present study, raising the idea that the chronic low-grade inflammation associated with a HCD and/or ageing drives development of something akin to an allergic type response. Certainly, it has previously been shown in humans that normal ageing can be associated with an increase in Th2 cells (24). What will be interesting now would be to further explore the targeting of IL-4 and IL-5 by ES-62 and the SMAs to determine whether we are witnessing a protective phenomenon similar to what happens in the allergic lung exposed to these reagents.

## Data availability statement

The original contributions presented in the study are included in the article/supplementary material. Further inquiries can be directed to the corresponding author.

## Ethics statement

The animal study was approved by University of Glasgow Animal Welfare and Ethical Review Board. The study was conducted in accordance with the local legislation and institutional requirements.

## Author contributions

MH: Conceptualization, Formal Analysis, Funding acquisition, Project administration, Supervision, Writing – original draft, Writing – review & editing. FL: Formal Analysis, Writing – review & editing, Investigation, Methodology. JC: Formal Analysis, Investigation, Methodology, Writing – review & editing. JD: Formal Analysis, Investigation, Methodology, Writing – review & editing. GB: Formal Analysis, Investigation, Methodology, Writing – review & editing. SB: Investigation, Writing – review & editing. GT: Investigation, Writing – review & editing. AM: Investigation, Writing – review & editing. CJS: Investigation, Methodology, Writing – review & editing. CS: Conceptualization, Formal Analysis, Funding acquisition, Writing – review & editing. WH: Conceptualization, Formal Analysis, Funding acquisition, Project administration, Supervision, Writing – original draft, Writing – review & editing.
